# Bioelectric device for effective biofilm inflammation management of dental implants

**DOI:** 10.1038/s41598-023-48205-2

**Published:** 2023-12-04

**Authors:** Jihyun Lee, Young Wook Kim

**Affiliations:** 1 Department of Periodontology, Ulsan University Hospital, College of Medicine, University of Ulsan, 877 Bangeojinsunhwando-ro, Dong-gu, Ulsan, 44033 Republic of Korea; 2 ProxiHealthcare Advanced Institute for Science and Technology (PAIST), Seoul, Republic of Korea

**Keywords:** Health care, Engineering

## Abstract

Dental implant inflammation is primarily caused by oral biofilms, which form within 8 h, particularly at 37 °C, thereby requiring diligent cleaning. Considering the complex management of dental implants, a novel technology based on the bioelectric effect (BE) to combat inflammation has emerged. A BE-integrated toothbrush was developed and clinically tested on patients with dental implants (N = 36). Our findings revealed a significant average plaque index reduction of 67% with BE technology compared with that at baseline (P < 0.05), whereas non-BE did not yield statistical significance even after 4 weeks of use (P > 0.05). The bleeding index demonstrated a 59% average reduction in all surfaces with BE technology (P < 0.05), whereas the non-BE group exhibited no significant change. Substantial reductions in total plaque and bleeding indices suggest that using BE toothbrushes can help effectively remove oral biofilms and treat bleeding symptoms.

## Introduction

Dental implants have contributed to better quality of life worldwide since their invention in 1978^1^. According to the American Academy of Implant Dentistry, over 2.5 million implant surgeries have been performed in the US (only one percent of the population with missing teeth)^2^. As life expectancy increases, dentists expect more people to receive dental implants. However, implants can cause a consistent immune reaction, as they are recognized as external materials, resulting in severe inflammation near the implant^3^. Particularly, when oral biofilms are established on implants, inflammation can be accelerated because the biofilms facilitate the immune reaction^4^. Inflammatory peri-implant disease is categorized into peri-implant mucositis and peri-implantitis. Peri-implant mucositis is a reversible inflammatory lesion of the peri-implant soft tissue, while peri-implantitis is a lesion that affects the supporting bone as well as the mucosa. Peri-implant mucositis is usually diagnosed by clinical signs of inflammation, such as bleeding on probing and/or suppuration. Severe peri-implantitis often requires invasive and time-consuming reoperation^5^. Therefore, removing oral biofilms is crucial to preventing implant failure^6^.

Effective removal of oral biofilms can be achieved by regular visits to dental clinics and the use of diverse dental devices at the same time^7^. Biofilms can grow within 8 h under normal oral conditions^8^. Regular cleaning of oral biofilms is essential. Toothbrushes are a classical and standard method for biofilm removal because they can directly remove the biofilm. However, oral inflammation typically occurs between the teeth and gingiva, a deep pocket area where the current bristles cannot reach^9^. Oral inflammation is significantly associated with systemic diseases, including stroke, Alzheimer’s disease, cardiovascular disease, diabetes, and hypertension^10,11^. Hence, effective cleaning of biofilms is critical. Owing to this limitation of current toothbrushes, the development of new technology for cleaning deep pocket areas is of great interest for advances in oral health.

Combinatorial treatment of biofilms using small amounts of electricity enhances the efficacy of biofilm removal; this is known as the bioelectric effect (BE)^12^. Our group focused on reducing electric voltage below 0.82 V, which is not induced electrolysis of media^13^. In previous studies, BE technology has demonstrated a significant reduction in biofilms as well as biocompatibility by showing non-electrolysis^14^. Based on this technology, we developed a BE-integrated toothbrush dedicated to cleaning oral biofilms that the bristle cannot reach^15^. The BE enables deep pocket biofilms to propagate through water-based media (salivary) electric current flow^16^.

Existing studies have focused on either validating the benefits of electric toothbrushes to a limited extent or presenting the differences between them and regular toothbrushes. However, there is a lack of thorough evaluation and analysis of the effective use of microcurrent toothbrushes, their actual impact on the teeth and gingiva, and user experience. To the best of our knowledge, this is the first study to investigate the effectiveness of microcurrent toothbrushes for dental implants.

In this study, we focused on evaluating the BE toothbrush for the efficacy of plaque (oral biofilm) removal and reduction of bleeding, which is a crucial indication of inflammation symptom management. The clinical trial was designed as a blind test with four dentistry visits. The performance was compared with that of a non-BE toothbrush in which the bristle and design of the device were identical. Quantitative analysis of the plaque and bleeding index was performed using statistical analyses (ANOVA, Chi-square test, and P-value analysis).

## Results

We conducted clinical trials in patients with dental implants. The quantitative analysis focused on bleeding (modified Sulcus Bleeding Index [mSBI]) and plaque index (modified Plaque Index [mPI]), as presented in Tables 1, 2.

**Table 1:** Quantitative measurement of mPI and mSBI under non-BE and BE conditions. “ALL” indicates the entire data analysis along the interproximal and lingual areas.

	mPI					mSBI				
	Initial		After 4 weeks		p-value	Initial		After 4 weeks		p-value
	AVG	Stdev	AVG	Stdev		AVG	Stdev	AVG	Stdev	
ALL										
Non-BE	0.70	0.35	0.76	0.42	0.344	0.42	0.29	0.50	0.3	0.229
BE	0.88	0.44	0.29	0.24	0.000	0.70	0.39	0.29	0.22	0.000
Interproximal										
Non-BE	0.83	0.50	0.85	0.57	0.988	0.36	0.34	0.52	0.39	0.012
BE	1.05	0.58	0.32	0.38	0.000	0.78	0.58	0.22	0.23	0.000
Lingual										
Non-BE	0.73	0.68	1.01	0.81	0.027	0.65	0.69	0.69	0.73	0.791
BE	0.77	0.53	0.45	0.53	0.005	0.81	0.58	0.52	0.53	0.026

**Table 2:** Normalization respected to the initial value.

	mPI					mSBI				
	Initial		After 4 weeks		p-value	Initial		After 4 weeks		p-value
	AVG	Stdev	AVG	Stdev		AVG	Stdev	AVG	Stdev	
ALL										
Non-BE	106%	50%	109%	60%	0.344	100%	69%	119%	71%	0.229
BE	100%	50%	33%	27%	0.000	100%	56%	41%	31%	0.000
Interproximal										
Non-BE	100%	60%	102%	69%	0.988	100%	94%	144%	108%	0.012
BE	100%	55%	30%	36%	0.000	100%	74%	28%	29%	0.000
Lingual										
Non-BE	100%	93%	138%	111%	0.027	100%	106%	106%	112%	0.791
BE	100%	69%	58%	69%	0.005	100%	72%	64%	65%	0.026

The study included 36 participants. There were no dropouts. The average age of the participants was 60.28 ± 8.28, with 20 males and 16 females. The time of implant placement ranged from 1 to 19 years (mean ± SD = 9.48 ± 3.88 years), and the time of implant loading ranged from 1 to 19 years (mean ± SD = 8.80 ± 3.97 years) (Osstem implant system, TS CA Fixture, Seoul, Korea). All were for metal abutments and gold crowns. Continuous variables were compared using the independent *t*-test, and categorical variables were compared using the Chi-square test.

**Figure 1:**
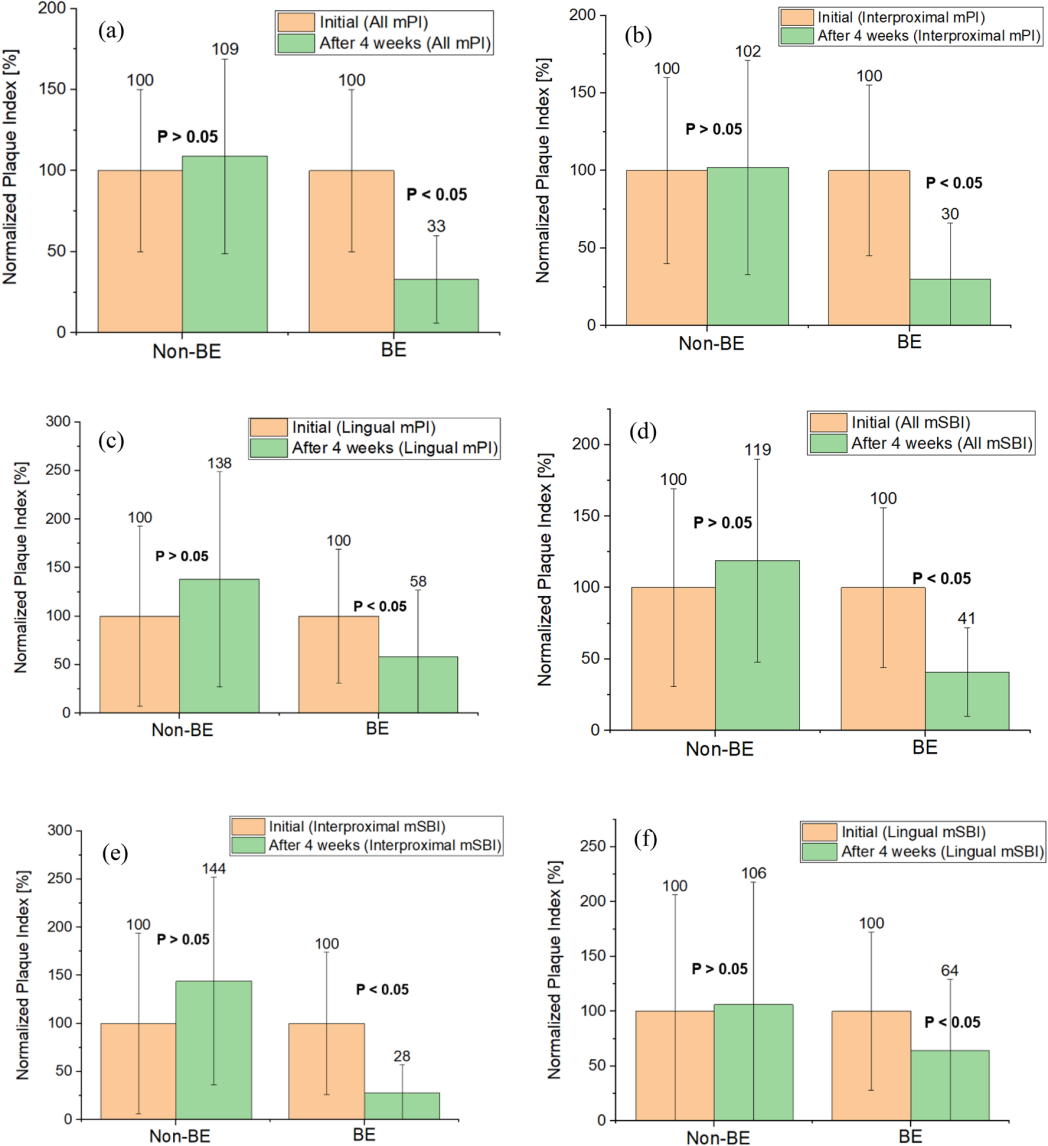
Normalized mPI and mSBI values compared to non-BE with BE (initial and after 4 weeks of use of non-BE (n = 18) or BE toothbrush (n = 18)). (**a**–**c**) Present mPI in all, interproximal, and lingual area measurements. Only the BE toothbrush shows statistical significance (ANOVA, P < 0.05). (**d**–**f**) Show mSBI in all, interproximal and lingual area data (average with standard deviation). The mSBI also only demonstrated statistical significance under the BE condition (ANOVA, P < 0.05).

**Figure 2:**
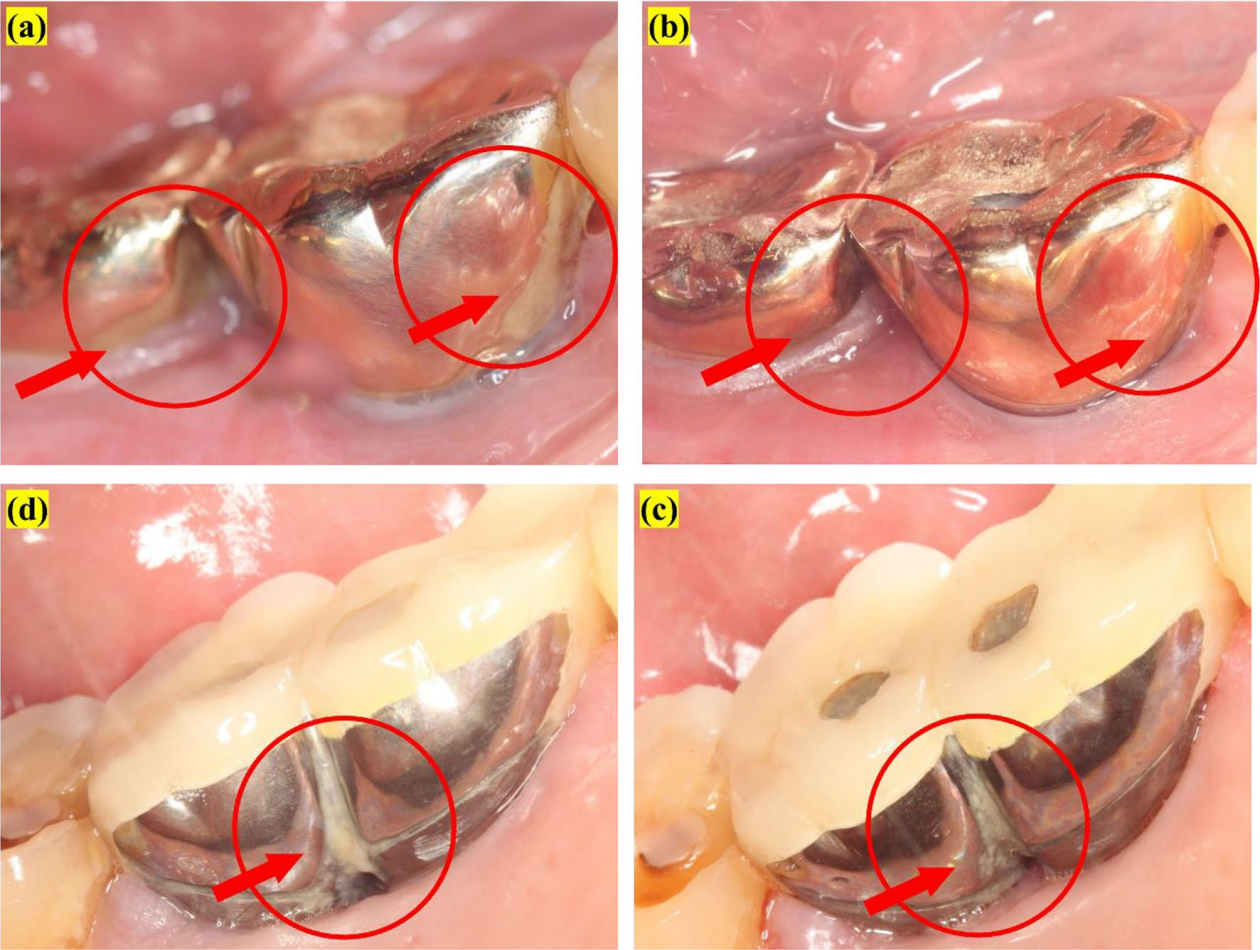
Representative images of mPI: (**a**) initial image, (**b**) after 4 weeks of use of BE toothbrush, (**c**) initial image, and (**d**) after 4 weeks of use of non-BE toothbrush. Under the BE toothbrush condition, a significant reduction of plaque has been observed (red circle).

The mPI and mSBI values were averaged for all surfaces, interproximal surfaces, and lingual surfaces. Each implant was measured at four locations, facial, mesiofacial, distofacial, and lingual. There was no statistically significant difference in the mPI and mSBI before and after brushing in the non-BE toothbrush group. However, there was a significant decrease in mPI and mSBI after brushing compared with before brushing in the BE toothbrush group (P < 0.05) (Tables 1, 2, Figs. 1, 2).

**Figure 3:**
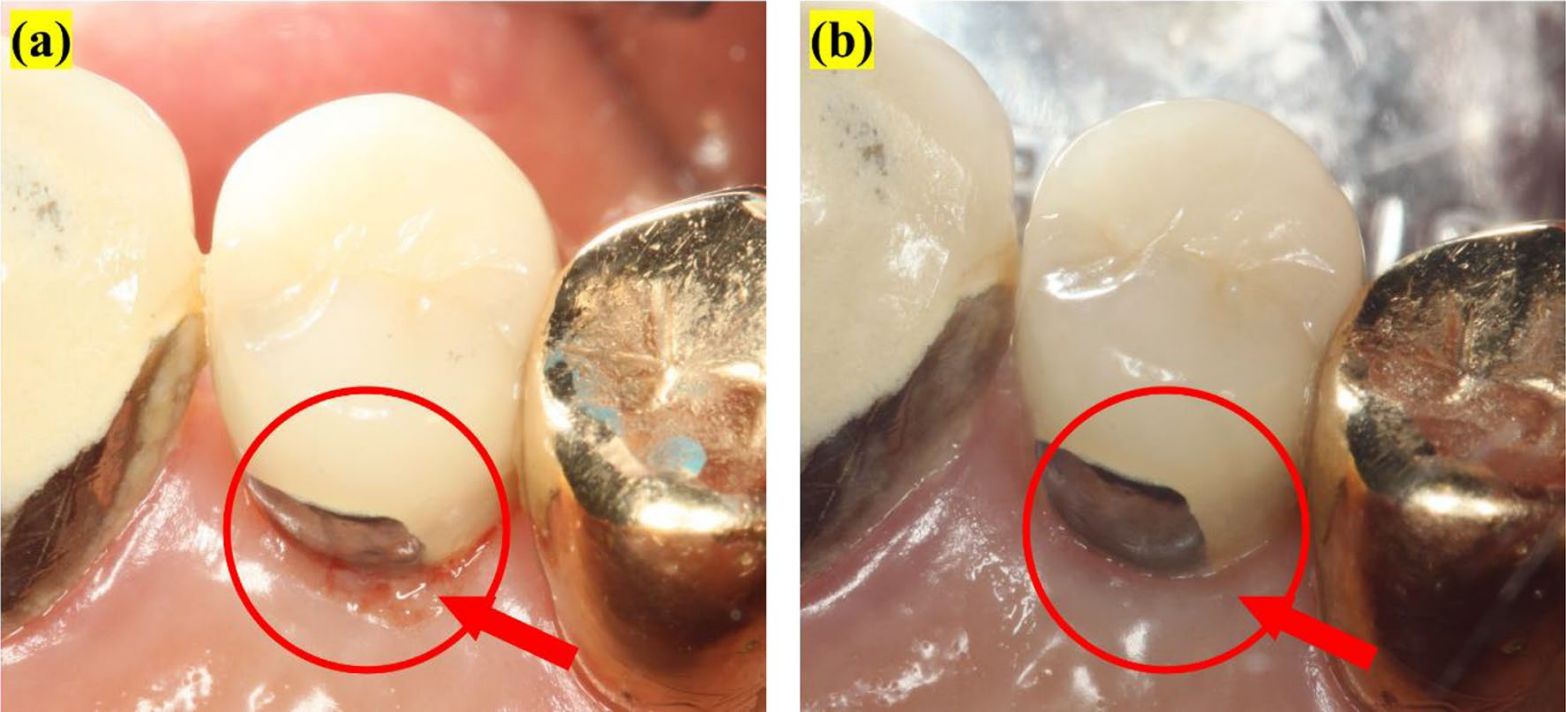
Representative images of mSBI: (**a**) initial image, (**b**) after 4 weeks of use of the BE toothbrush. Under the BE toothbrush condition, a significant reduction of the bleeding index has been observed (red circle).

Figures 2 and 3 present representative clinical photographs of a participant after using either the BE or non-BE toothbrush for 4 weeks. Reduction in bleeding and gingival redness was observed on the lingual surface of the implant. Figure 2 shows a reduction in plaque on the interproximal surface where the bristles had limited reach.

There was no significant carry-over effect for whether the washout period was adequate and no significant period effect for whether exposure to the toothbrush made a difference (P > 0.05).

A significant reduction in mPI and mSBI was observed only when the BE toothbrush was used. Under BE, mPI was reduced by 67% compared with that under non-BE, which was not statistically significant. A non-BE toothbrush can only remove plaques where the bristle reaches. There was no significant difference after 4 weeks of use from the initial period. However, BE toothbrushes can remove plaque based on the principles of BE. BE significantly reduced mPI (Fig. 1). There were a total of 36 volunteers for this clinical trial, and the number of BE and non-BE volunteers was 18 each.

The mSBI demonstrated that the BE toothbrush only showed a significant reduction compared with no difference under non-BE conditions (Figs. 1 and 3). As bleeding is induced by oral biofilm inflammation, BE can reduce bleeding through effective plaque reduction.

A more effective reduction in both mSBI and mPI has been demonstrated in the interproximal area, where a deep pocket is located, and the bristle can hardly reach there. Therefore, the efficacy of BE for mSBI and mPI can be observed more precisely.

Based on clinical trials, BE significantly reduced total plaque and bleeding symptoms.

## Discussion

Our findings demonstrated that the use of BE toothbrushes led to significant reductions in the mPI and mSBI scores. This enhanced effect was also significant in natural teeth without metal restorations in previous studies, confirming the bioelectric effect of propagating voltages, currents, and electric fields to weaken the gingival bacterial film. This study included participants who met the criteria for regular maintenance checks and excluded those with poor oral hygiene because previous studies have shown that individual differences in brushing ability may have affected the results^16^. The selection of patients with good brushing skills and a longer duration of BE toothbrush use (2 weeks in the previous study and 4 weeks in this study) may have contributed to the significant reductions in mPI and mSBI. Moreover, since the prosthesis was a gold crown, the plaque would have been more visible, and the timer to ensure the recommended brushing time ensured that participants brushed for at least two and a half minutes, likely contributing to the positive effect.

Taking these variables into consideration is crucial, as better plaque removal achieved with the current toothbrush may be attributed to the Hawthorne effect^17^, where participants alter their behavior owing to being observed in a study, or the novelty effect^18^, in which the use of a new toothbrush temporarily boosts motivation and willingness to use the product.

However, despite the good brushing ability, there was no difference between pre- and post-intervention when using a manual toothbrush. This suggests that the implant prosthesis was not easy to clean, and because 51 of the 54 examined implants (94.4%) were in the molar region, rendering it difficult for the bristles to reach. Consequently, BE toothbrushes are effective on interproximal and lingual surfaces that toothbrushes cannot reach, even on implant prostheses with complex structures (Tables 1, 2). The ages of participants ranged from 36 to 74, with a mean age of 60.28 ± 8.28. The BE toothbrushes are beneficial for geriatric patients with impaired manual skills.

Dental implants, such as teeth, are at risk of colonization by biofilms that cause inflammation in the supporting tissues. Failed implants are associated with microbial colonization^19^. The application of antimicrobials, as proposed to date to control biofilms, can create secondary problems. To address these issues, new highly controllable biofilm control technologies are required. Del Pozo et al. reported that different microbial species respond to different types of antimicrobials and electrical currents to different degrees, and control appears to be slow in mixed culture biofilms containing many species of microorganisms^20^. Microcurrent technology is another method of controlling microbial adhesion. We propose that alternating current (AC) and direct current (DC) electrical signals could be new alternatives for effectively managing biofilms^12^. A simpler approach to preventing inflammation could involve using electricity alone for control without the need for antibiotics^12^.

*Porphyromonas gingivalis* is a key pathogen in periodontal disease that plays a role in the development and progression of periodontitis and peri-implantitis^21^. Therefore, developing new therapies to effectively control P. gingivalis-associated biofilm infections is essential. Various studies have demonstrated that electric current can substantially inhibit microbial biofilms, defined as an “electrical effect”^22,23^. Zou et al. showed that constant DCs can inhibit biofilm formation by *P. gingivalis* in a time- and intensity-dependent manner^24^, and 1000 μA DCs can kill *P. gingivalis* and *P. intermedia* by promoting reactive oxygen species overproduction in vitro, suggesting that low-intensity DCs may be a promising approach to treat periodontal infections^25^.

Peri-implantitis is a multifactorial disease with many triggering factors that cannot be resolved by biofilm control alone. Controlled systemic diseases, ideal implant placement positions, and well-formed occlusions are essential. This study demonstrated its effectiveness in peri-implant mucositis; however, a follow-up, long-term study is needed to confirm its effectiveness in peri-implantitis. In future studies, we will further investigate how microcurrents work at the cellular and tissue levels in inflammatory responses by evaluating cytokine levels. Good oral hygiene is crucial for the prevention and treatment of peri-implantitis. People with periodontal disease need a regular periodontal maintenance program, as periodontal disease can be prevented by regularly reducing bacterial counts with supportive periodontal therapy. The progression to peri-implantitis is expected to be prevented by weakening the structure of the oral biofilm and controlling bacterial counts with a BE toothbrush.

Löe’s clinical research has established that gingivitis occurs when the number of plaques increases^26^. Similarly, in a study where patients with intraoral implants stopped brushing for 3 weeks and were monitored, inflammation was observed in the soft tissues around the implants and the expected plaque buildup^27^. Peri-implant mucositis is primarily caused by dental plaque, and the host’s immune response can vary depending on individual circumstances, such as smoking, diabetes, and radiation therapy. Peri-implant mucositis is defined as the inflammation of the mucous membrane around an implant, which can be reversed with improved oral hygiene, as in the case of gingivitis.

The advent of osseointegrated dental implants as a restorative treatment option to replace lost teeth has also introduced new artificial surfaces into the mouth, on which oral bacteria can form biofilms. Despite the large number of dental implants placed, dentists are not familiar with their aftercare, and there is no consensus regarding the treatment of peri-implant disease^28^. Early intervention with effective removal of microbial biofilms is known to prevent further progression to peri-implantitis^29^.

Microbial biofilms in the mouth are involved in the etiology of various oral conditions, including tooth decay, periodontal and endodontic diseases, bad breath, denture stomatitis, candidiasis, and dental implant failure^30^. Current treatment techniques include periodic mechanical removal of oral microbial biofilms and maintenance of therapeutic concentrations of antimicrobials in the mouth, both of which have limitations. Therefore, the development of alternative antimicrobial treatment strategies is vital to the advancement of methods to control oral microbial growth.

We previously reported a significant decrease in the gingival index on the interproximal surfaces, which is an area that is difficult for bristles to reach, in a group that used a toothbrush with bioelectric-microcurrent technology, such as bioelectric current^16^. While previous research focused on the mechanisms of direct or alternating current alone, this technology combines the signals from these two modalities to maximize their effectiveness. When direct and alternating currents are simultaneously applied to a biofilm, the metabolic stress on the biofilm increases rapidly, which induces the biofilm to drop off the surface^31,32^.

## Materials and methods

### Bioelectric device and electrical signal for BE

**Figure 4:**
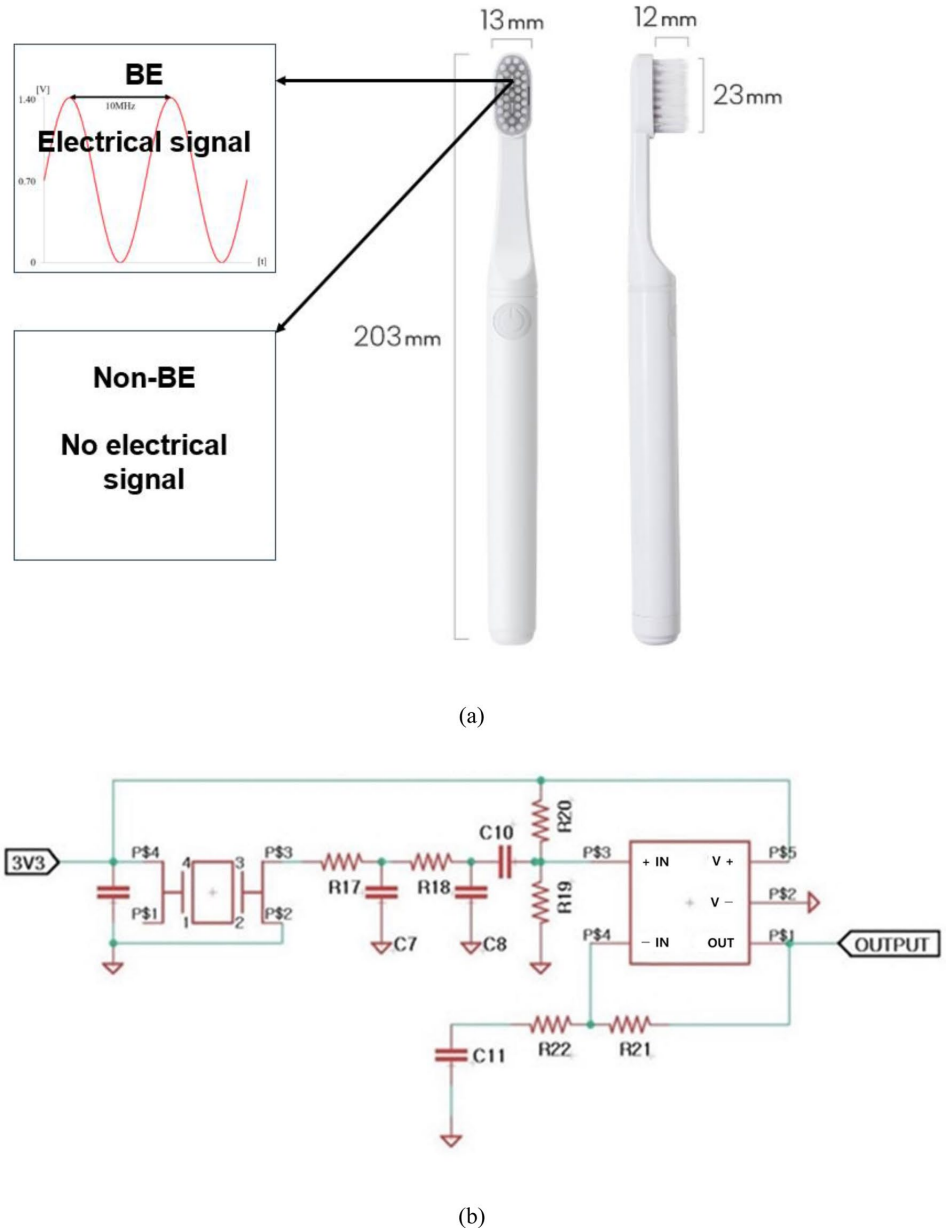
(**a**) Schematics of BE & non-BE toothbrush condition. Identical shape and bristles, but the presence or absence of the BE electrical signal. (**b**) schematic of embedded electronic system in toothbrush for generating the BE signal.

The electrical input comprised a 0.7 V amplitude sinusoidal AC at a frequency of 10 MHz with 0.7 V of a DC offset. The frequency was selected based on a previous work^15,16^, and the DC offset was defined to avoid the electrolysis threshold (below 0.82 V) in bacterial growth media, as summarized in Table 3. The electrolysis thresholds for water and bacterial growth media are different, and this threshold was chosen because bacterial biofilms were the target of this study. A schematic of the electrical signal is shown in Fig. 4a. An electronic circuit embedded in the toothbrush was developed as shown in Fig. 4b^14,15,33^.

**Table 3:** Details of the bioelectric effect.

Contents	Details	Comments
Intensity	0.70 V	Below water electrolysis 0.82 V
Frequency	10 MHz	Effective frequency
Composition (AC:DC)	1:1	Effective biofilm treatment

### Clinical trials

This was a randomized, double-blind, crossover clinical study in which participants received one test brush (BE), one control brush (non-BE), and one toothpaste (Tromatz toothpaste, Sungwon Co., Ltd., Goyang, Korea). Both the test brush (BE, proxyhealthcare, Ulsan, Korea) and the control brush (non-BE, proxyhealthcare, Ulsan, Korea) are soft and multi-tufted. The bristles are 12 mm long and 0.15 mm in diameter. It is made from Polybutylene terephthalate (Fig. 4).

**Figure 5:**
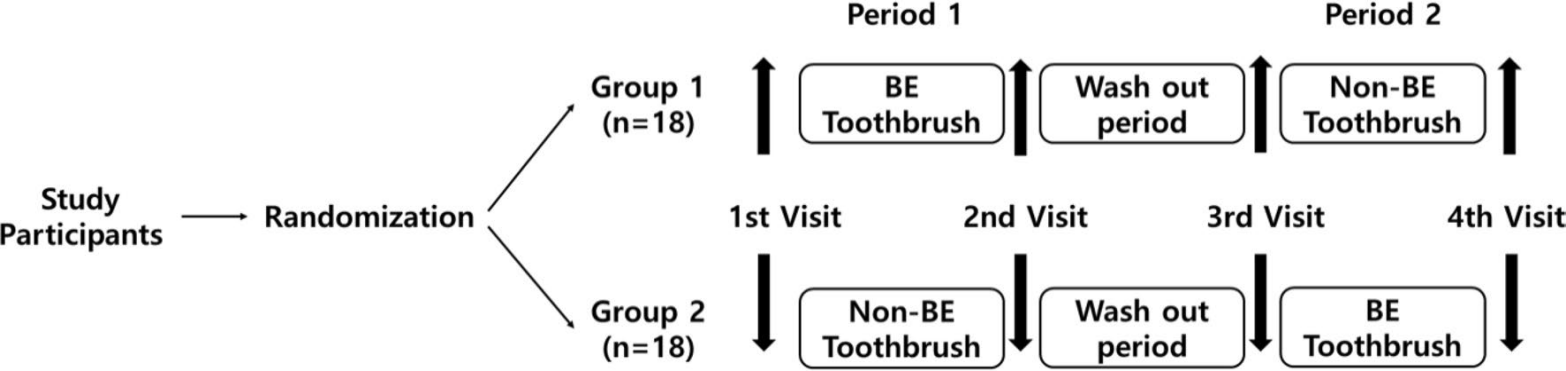
Flow chart of the clinical study (subjects were randomly assigned to the groups).

Toothpaste was used because it contains sodium chloride, which activates ionic movement and increases the microcurrent effect. Participants were randomized using a random number table and their assigned toothbrush (BE, 4 weeks; non-BE, 4 weeks) for 4 weeks, followed by a 2-week washout period and four visits (Fig. 5). A research assistant performed the randomization, enrolled the participants, and distributed toothbrushes to blind the researchers.

We decided not to control the frequency, time, or method of brushing but to allow individuals to continue their usual routine and use their usual toothbrush during the 2-week pause. This was done by brushing the teeth with a BE toothbrush to remove any impact on the oral flora and returning the patient to their previous state. During the study, the participants were prohibited from using any oral hygiene products other than the toothbrush provided.

### Participants

The study was conducted with 36 volunteers from September 2022 to May 2023, who provided informed consent, and was approved by the Institutional Review Board of Ulsan National University (IRB protocol No:2022-06-012) and conducted in accordance with the Declaration of Helsinki of 1975, as revised in 2013. The inclusion and exclusion criteria for study participants are listed in Table 4.

**Table 4:** Inclusion and exclusion criteria.

Inclusion criteria	Exclusion criteria
19–75 years old	Patients with uncontrolled systemic disease
No history periodontal treatment within 3 months	Poor oral hygiene and compliance
No history of medication antibiotics in the past 3 months	Fixed or removable orthotic appliance
Implant prosthetics with visualization of plaque (Ex. Gold crown, metal abutment /c supragingival margin)	Severe periodontitis in adjacent teeth
Bleeding on probing, no periodontal pockets, and no radiographic peri-implant bone loss	Person who withdraws informed consent as a participant

The mPI and mSBI^34^ were measured at each visit. The mPI for dental implant scales was as follows (0 = no plaque, 1 = plaque at the cervical margin difficult to see, 2 = plaque can be seen by the naked eye, 3 = abundance of soft matter). The mSBI for dental implant scales is as follows (0 = no bleeding when the periodontal probe is passed along the gingival margin; 1 = isolated bleeding spot visible; 2 = blood forms a confluent red line on the gingival margin; 3 = heavy or profuse bleeding), as summarized in Table 5. Assessment and recording of mPI and mSBI were performed by the same investigator, a periodontist, to reduce inter-investigator error.

**Table 5:** Details of mPI and mSBI.

mPI (modified Plaque Index)	mSBI (modified Sulcus Bleeding Index)
– Score 0 : No detection of plaque – Score 1 : Plaque only recognized by running a probe across the smooth marginal surface of the implant. Implants covered by titanium spray in this area always score 1 – Score 2 : Plaque can be seen by the naked eye – Score 3 : Abundance of soft matter	– Score 0 : No bleeding when a periodontal probe is passed along the gingival margin adjacent to the implant – Score 1 : Isolated bleeding spots visible – Score 2 : Blood forms a confluent red line on margin – Score 3 : Heavy or profuse bleeding
PI for a tooth = scores of 4 areas/4 PI for individual = total scores/no. of teeth examined PI for group = total scores/no. of individuals	SBI = total scores of 4 areas/ 4
Areas examined: distofacial, facial, mesio-facial, lingual***	Areas examined: labial, lingual marginal gingiva, mesial, and distal papillary gingiva

### Safety assessment

The safety of BE toothbrushes is well established, and our previous studies have shown that they are not dangerous. However, if damage to the gingiva is observed due to the patient's brushing habits or if the patient complains of discomfort with the product, its usage will be stopped, and the patient will be excluded from the study.

## Data Availability

The datasets used and/or analyzed during the current study available from the corresponding author on reasonable request.
